# Post-viral idiopathic purpura fulminans is associated with inherited thrombophilia and anti-cardiolipin antibodies

**DOI:** 10.3389/fped.2023.1197795

**Published:** 2023-05-30

**Authors:** A. Theron, S. Ayadi, E. Boissier, O. Dautremay, J.-F. Schved, N. Sirvent, I. Diaz, G. Captier, C. Biron-Andreani, E. Jeziorski

**Affiliations:** ^1^ Department of Pediatric Oncology and Hematology, CHU de Montpellier, University of Montpellier, Montpellier, France; ^2^ Hemophilia Treatment Center, Montpellier, France; ^3^ IRMB, University of Montpellier, INSERM, Montpellier, France; ^4^ Laboratory of Hematology, University Hospital, Nantes, France; ^5^ Biology Laboratory, Charleville-Mézières, France; ^6^ Department of Biological Hematology, CHU de Montpellier, University of Montpellier, Montpellier, France; ^7^ Department of Plastic, Reconstructive and Aesthetic Surgery, CHU de Montpellier, University of Montpellier, Montpellier, France; ^8^ LIRMM, CNRS-University of Montpellier, Montpellier, France; ^9^ Department of Pediatric Infectious Diseases and Immunology, CHU de Montpellier, University of Montpellier, Montpellier, France; ^10^ PCCEI, CeRéMAIA, CHU de Montpellier, University Montpellier, Montpellier, France

**Keywords:** purpura fulminans, thrombophilia, anti-cardiolipin antibodies, child, chickenpox

## Abstract

**Introduction:**

Idiopathic purpura fulminans (IPF) is a rare and severe coagulation disorder, associated with transient anti-protein S (anti-PS) antibodies in the context of post-viral infection such as varicella. Anti-protein S antibodies are frequently found in the context of varicella, in contrast with the rarity of IPF. Other factors such as anti-phospholipid antibodies (APL) and inherited thrombophilia may be associated with severe vascular complication.

**Method:**

This is an ancillary study of a French multicenter retrospective series and systematic review of literature. We analyzed patients who were tested for inherited thrombophilia, namely antithrombin, protein C, protein S deficiency; prothrombin gene G20210A polymorphism (FII:G20210A),Factor V R506Q polymorphism (FV:R506Q); and/or for APL (lupus anticoagulant (LA), anti-cardiolipin antibodies (ACL), or anti-beta 2-GPI antibodies (Aβ2GP1).

**Results:**

Among the 25 patients tested for inherited thrombophilia, 7 (28%) had positive results. Three had FV R506Q, two FII:G20210A, one compound heterozygote FV:R506Q associated to FII:G20210A, and one protein C deficiency. APL testing was performed in 32 patients. It was positive in 19 patients (59%): 17 ACL (53%), 5 LA (16%), 4 Aβ2GP1 (13%). The risk of severe complications was not associated with presence of inherited thrombophilia or APL presence, with RR: 0.8 [95% CI: 0.37–1.71], *p* = 1 and *RR*: 0.7 [95% CI: 0.33–1.51], *p* = 0.39, respectively. We found a high prevalence of inherited thrombophilia or APL in a population of patients with IPF. However, we do not find an association with the occurrence of severe vascular complications or venous thromboembolism.

## Introduction

Idiopathic purpura fulminans (IPF) is a rare and severe prothrombotic disorder, affecting children after a primary infection by common infectious entities such as the varicella-zoster virus (VZV) or human herpes virus 6 (HHV6) (1). This coagulation disorder is related to transient autoantibodies directed against the coagulation inhibitor protein S (PS) (2). The putative mechanism involves antigenic mimicry between the virus and PS (3). These antibodies may induce a significant decrease in PS and profound disruption of the protein C (PC)/PS regulatory pathway. Deregulation of the PC/PS pathway leads to a state of hypercoagulability that causes thrombotic lesions affecting mainly the lower limbs. In the most severe forms, extensive skin necrosis and distal amputations are observed (1–4). Anti-protein S antibodies are frequently found in the context of varicella, in contrast with the rarity of IPF (4, 5). The existence of anti-PS antibodies, therefore, may not be the only condition leading to PFI. We hypothesized that cofactors such as inherited thrombophilia or other immune disorder could be involved (6–8). Interestingly in the literature, the presence of antiphospholipid antibodies (APL) is often noted in cases or series of idiopathic purpura fulminans (5, 9).

Our main objective was, therefore, to determine the frequency of inherited thrombophilia and APL in patients with IPF.

## Methods

### Study design

This is an ancillary study of our French retrospective study and review of the literature (10). All the methodological details of the patient recruitment and data collection are detailed in this previous publication. The inclusion criteria were patients aged less than 18 years, presenting a clinical presentation of idiopathic purpura fulminans in a post-infectious context and with evidence of a transient protein S deficiency. All data were collected retrospectively from the patient files for the patients of the retrospective French cohort and from the literature for the patients of the historical series.

For this study, we focus on patients who were tested for APL and/or thrombophilia. APL was assayed according to ISTH recommendations (11), including assays for lupus anticoagulant (LA) anti-cardiolipin antibodies (ACL) (IgG or IgM), and anti-beta 2-GPI antibodies (Aβ2GP1) (IgG or IgM). Inherited thrombophilia was defined by the presence of any of the following: deficiency in antithrombin, PC or PS; occurrence of a G20210A polymorphism in the prothrombin gene (FII:G20210A) or a R506Q polymorphism in the Factor V gene (FV:R506Q). The diagnosis of PC, PS or AT deficiency is considered if there is a level below the norm at a distance from the acute episode with, if available, confirmation by genetic analysis or family study. The methods for measuring protein S, protein C, antithrombin, and antiphospholipid antibodies may vary from one patient to another because of the multicenter and retrospective nature of the study. We have tried to respect each time the reference linked to each technique for the interpretation of the results.

The main study was approved by the institutional review board of the Montpellier University Hospital on behalf of all participating centers, and it complied with the regulations of the Declaration of Helsinki. All genetic analyses were performed with written consent of the parents in accordance with the French legislation for cases in the French registry. The main study has been registered on clinicaltrial.gov under number NCT04845113.

### Statistical analysis

The continuous variables are presented as medians with the minimum and maximum values, and binary variables as the ratio of the total population. Distal necrosis leading to amputation and skin necrosis requiring skin grafting were considered to be severe complications. We used Fisher’s exact test and calculated relative risk to study APL or thrombophilia association with patient outcomes. The statistical analyses were performed using GraphPad Prism version 9 for Windows software (GraphPad Software, San Diego, California, USA).

## Results

### Description of the population

The series includes forty-four patients, fourteen patients from the French series, and thirty patients from the literature review cases.

The median age at diagnosis was 4.9 years (1.5–11), with a slight male predominance of 24/44 (55%). The diagnosis of viral infection was confirmed in 39/44 patients: 35 cases of varicella (80%) and four cases of HHV6 (9%). PS deficiency was confirmed in all patients, with a median PS activity of 4% (1–28), a median free PS of 1% (1–16), and a median total PS of 5% (1–62). Anti-PS antibodies were tested and detected in 23 patients (52%). Regarding complications, 12 (27%) patients underwent amputation, 11 (25%) underwent skin grafting, and 13 (30%) had deep vein thrombosis (details in Table 1).

**Table 1:** Patient characteristics, type of virus, biology at diagnosis, and outcomes.

	All patients (*n* = 44)	
	No. (%)	Median (range)
**Patient characteristics**		
Age in years, median (range)		4.9 (1.5–11)
Male sex	24 (55)	
**Virus**		
HHV-6	4 (9)	
VZV	35 (77)	
Unknown	5 (13)	
**Biology at diagnosis**		
PS activity, % median (range)		4 (1–28)
PS free antigen, %		1 (1–16)
PS total antigen, %		5 (1–62)
Anti-PS antibodies	23 (52)	
**Outcomes**		
Amputations, *n* (%)	12 (27)	
Skin necrosis with graft	11 (25)	
Venous thromboembolism	13 (29)	

VZV, varicella-zoster virus; HHV-6, human herpesvirus 6.

### Antiphospholipid antibodies

APL was found in 19 of 32 (59%) patients tested 17 ACL (53%), five LA (16%), four Aβ2GP1 (12.5%). Some patients had various combinations of antibodies, LA + ACL, *n* = 2; ACL + Aβ2GP1, *n* = 3; and LA + ACL + Aβ2GP1, *n* = 1 (details in Table 2). 6 (32%) patients with a positive APL present a severe complication and 5 (26%) present venous thrombosis. A positive APL test at the time of the IPF was not associated with severe complications, *RR*: 0.7 [95% CI: 0.33–1.51], *p* = 0.39, or venous thrombosis, *RR*: 0.9 [95% CI: 0.47–1.64], *p* = 1.

**Table 2:** Prevalence of inherited thrombophilia and antiphospholipid antibodies in the two-case series.

	Case series	Literature review cases	Total
Inherited thrombophilia testing, *n*	11	14	25
Inherited thrombophilia, *n* (%)	3 (27)	4* (28)	7* (28)
Factor V R506Q polymorphism, *n* (%)	2 (18)	2 (14)	4 (16)
Prothrombin G20210A ism, *n* (%)	1 (9)	2 (14)	3 (12)
Protein C deficiency, *n* (%)	0	1 (7)	1 (4)
Antithrombin deficiency, *n* (%)	0	0	0
Protein S deficiency, *n* (%)	0	0	0
Antiphospholipid testing^†^, *n*	9	23	32
Positive antiphospholipid (aPL), *n* (%)	5^†^ (55)	14^†^ (61)	19^†^ (59)
Lupus anticoagulant (LA), *n* (%)	3 (33)	2 (9)	5 (16)
Anti-cardiolipin antibodies (ACL), *n* (%)	3 (33)	14 (61)	17 (53)
Anti-beta 2-GPI antibodies (β2GP1), *n* (%)	2 (22)	2 (9)	4 (13)

* 1 case had both Factor V and II polymorphism.

^†^ Several tests were positive in some cases: LA + ACL, *n* = 2; ACL + β2GP1, *n* = 3; LA + ACL + β2GP1, *n* = 1.

### Inherited thrombophilia

Seven (28%) out of the 25 patients tested for thrombophilia had positive biological ‘markers: three heterozygous FV:R506Q, one homozygous FV:R506Q, three FII:G20210A, and one inherited familial PC deficiency (details in Table 2). In one case, the patient had both heterozygous FV:R506Q and FII:G20210A. All patients corrected their protein S and antithrombin levels at a distance from the event. In one patient, a protein C deficiency was confirmed in the context of a known and explored family deficiency. 4 (57%) patients with inherited thrombophilia present a severe complication and 3 (48%) present venous thrombosis. Severe complications and venous thrombosis were not associated with inherited thrombophilia (*RR*: 0.8 [95% *CI*: 0.37–1.71], *p* = 1; *RR*: 1.2 [95% *CI*: 0.40–3.62], *p* = 1). None of the patients with inherited thrombophilia had APL.

## Discussion

Our study find a high prevalence of APL, especially ACL associated with the diagnosis of IPF. ACL is known to be associated with thrombosis in antiphospholipid antibody syndrome (12–15) and has also been well described as transient and inconsequential in post-infectious settings in children (16, 17). In an extensive literature review of post-infectious APL, varicella was the main viral cause in pediatric cases (18). Our results are similar to previous studies, which found an excess prevalence of APL in children with varicella (4, 5). We found a majority of ACL as described in the context of transient post-infectious APL in children (17, 18). The presence of APL is not always associated with thrombosis in the post-infectious context; for example, the pro-thrombotic impact of APL is frequently being debated in the context of the COVID-19 pandemic, and it would appear that only APL pre-existing COVID-19 infection is responsible for a thrombotic excess risk (19). Concerning the frequency of APL in children, there are few clear data, one study suggests 11% of APL in apparently healthy children (20) and another study finds 30% of APL in children with upper airway infection (21). In a series of 491 children with connective tissue disorder, 71 had APL positive test and 11 had venous thrombosis (22).

In our series, the prevalence (28%) of markers associated to an inherited thrombophilia in patients with IPF is higher than in the general population (Table 3) (23–26). This prevalence raises the question of a possible link between hereditary thrombophilia and IPF. We could not find an association with thrombotic outcomes (distal necrosis leading to amputation, skin necrosis, or venous thromboembolism). Concerning distal necrosis leading to amputations and skin necrosis, these lesions appear to be related to arterial or capillary thrombosis. Conventional thrombophilia is known to be risk factors for venous thrombosis but not for arterial events.

**Table 3:** Prevalence of inherited thrombophilia in the general population (25–27), in children with venous thromboembolic disease (23, 24), and in patients with idiopathic purpura fulminans.

	General population	Children with venous thromboembolic disease	Children with idiopathic purpura fulminans
Antithrombin deficiency	0.02%–0.2%	1%	0
Protein C deficiency	0.2%–0.5%	1%	4%
Protein S deficiency	Unknown	1%	0
Factor V R506Q mutation	3%–7%	5%–10%	16%
Prothrombin G20210A mutation	2%	2%–3%	12%

Our study has limitations due to its retrospective design. Nevertheless, the rarity of IPF syndrome, knowledge about anti-PS antibodies, and the epidemiological data about acquired and inherited thrombophilia associated with known mechanisms of coagulation dysregulation support a hypothesis of a “domino effect” responsible for a pro-coagulation imbalance. Thrombophilia could promote the development of IPF; a similar mechanism has been suggested in cases of warfarin-induced necrosis in which the existence of a PC or PS deficiency predisposes to extensive skin necrosis at the time of drug introduction (6–8). Larger studies including early arterial and capillary lesions at the time of IPF episodes may be the key to understanding the pathophysiology of this rare and atypical vascular pathology.

In conclusion, our study found a high prevalence of inherited thrombophilia in a series of patients with IPF by acquired PS deficiency but the role of inherited thrombophilia in IPF remains unclear. However, we did not find an association between this condition and the occurrence of severe vascular complications or venous thromboembolism in IPF. Further studies are required, and prospective data collection may be a better way to improve the knowledge of biological factors associated with PS antibodies and their tragic complications in children.

## Data Availability

The raw data supporting the conclusions of this article will be made available by the authors, without undue reservation.
